# Synthesis and Properties of Water-Soluble Blue-Emitting Mn-Alloyed CdTe Quantum Dots

**DOI:** 10.1186/s11671-018-2529-y

**Published:** 2018-05-02

**Authors:** Olena Tynkevych, Volodymyr Karavan, Igor Vorona, Svitlana Filonenko, Yuriy Khalavka

**Affiliations:** 1 Yuriy Fedkovych Chernivtsi National University, Kotsiubynsky Str. 2, Chernivtsi, 58012 Ukraine; 2 The Scientific Center of Preventive Toxicology, Food and Chemical Safety, Fedkovycha Str. 30, Chernivtsi, 58000 Ukraine; 3 V. Lashkaryov Institute of Semiconductor Physics of National Academy of Sciences of Ukraine, 45, Pr. Nauky, Kyiv, 03028 Ukraine; 4 L. V. Pisarzhevskii Institute of Physical Chemistry of National Academy of Sciences of Ukraine, 31, Pr. Nauky, Kyiv, 03028 Ukraine; 5 Aalto University School of Science, P.O. Box 12200, FI-00076 Aalto, Finland

**Keywords:** Quantum dots, Cadmium telluride, Manganese, Alloying, Thioglycolic acid

## Abstract

In this work, we prepared CdTe quantum dots, and series of Cd_1-x_Mn_x_Te-alloyed quantum dots with narrow size distribution by an ion-exchange reaction in water solution. We found that the photoluminescence peaks are shifted to higher energies with the increasing Mn^2+^ content. So far, this is the first report of blue-emitting CdTe-based quantum dots. By means of cyclic voltammetry, we detected features of electrochemical activity of manganese energy levels formed inside the Cd_1-x_Mn_x_Te-alloyed quantum dot band gap. This allowed us to estimate their energy position. We also demonstrate paramagnetic behavior for Cd_1-x_Mn_x_Te-alloyed quantum dots which confirmed the successful ion-exchange reaction.

**Electronic supplementary material:**

The online version of this article (10.1186/s11671-018-2529-y) contains supplementary material, which is available to authorized users.

## Background

The optical properties of quantum dots (QDs) can be manipulated by doping/alloying through designing the composition. Consequently, the possibility of control over the incorporation of doping/alloying elements into QDs lattices plays an important role for a large number of applications. In particular, Mn-alloyed QDs are one of the most promising materials for fluorescence sensing and magnetic resonance imaging. Consequently, synthesis techniques have already been developed for obtaining Mn^2+^-doped/alloyed ZnS, ZnSe, CdSe, CdS, and core/shell CdTe/CdS QDs [1–6]. There is also a number of works that describe the capabilities of Mn^2+^- and Zn^2+^-doped/alloyed CdTe QDs synthesis [7–10]. Obtaining of such compositions in water solution is strongly pH-dependent that makes doping difficult. Cheng et al. showed that alkaline medium hindered the nucleation and growth of Zn-alloyed CdTe QDs [10]. Additionally, there is a high probability of appearance of Zn(OH)_2_ on the surface of QDs that inhibits the growth of initial QDs by forming ZnO shell on the surface of QDs. Similar processes take place in an attempt to prepare a Mn-alloyed CdSe QDs in alkaline aqueous solution [11]. In this case, Mn^2+^ solvation occurs instead of the ion exchange of Cd^2+^ by Mn^2+^. On the other hand, the difference of MnTe and CdTe solubility constant indicates ineffective substitution reaction between Mn^2+^ and CdTe [12].

In this work, we apply the synthetic procedure described in our previous work [13] for the synthesis of Cd_1−x_Mn_x_Te-alloyed QDs. To ensure a successful Mn-alloying process, the optimum neutral pH conditions were chosen. Such approach eliminated the formation of manganese hydroxide during the synthesis that allowed us to obtain blue-emitting Cd_1−x_Mn_x_Te-alloyed QDs by an ion-exchange reaction. The systematic studies of their optical and electrochemical properties enable a better understanding of the changes in the band structure during the transformation of CdTe QDs into Cd_1−x_Mn_x_Te-alloyed QDs.

## Methods

### Synthesis of CdTe and  Cd_1-x_Mn_x_Te-alloyed QDs

Water-soluble thioglycolic acid-stabilized CdTe and Cd_1-x_Mn_x_Te-alloyed QDs were synthesized according to our previously reported modified three-step method [13]. Firstly, CdTe nanoclusters were synthesized using a facile room temperature method [14] with thioglycolic acid as a stabilizer. The obtained CdTe nanoclusters were divided into six different 50 ml aliquots. Secondly, obtained colloidal solutions of CdTe nanoclusters were subjected to Mn^2+^ alloying due to ion exchange process with different amounts of MnSO_4_ salt in 50 ml aliquots solutions under sonication. The concentrations of Mn^2+^ ions added were 1, 5, 10, 15, and 20% of Cd^2+^ content in the reaction mixtures (RM). Also, one aliquot of pure CdTe nanoclusters was used as a control. The final step was a thermal treatment possess by microwave heating in the microwave oven at 700 W for 3 min.

### Material Characterization and Measurement Procedures

Absorption and photoluminescence (PL) spectra were measured at room temperature by the OceanOptics USB-2000 spectrophotometer. The cyclic voltammograms (CV) were recorded using a computer-controlled Potentiostat/Galvanostat “ΠИ-50-1.” A three-electrode system consisted of a platinum working electrode, a glassy carbon counter electrode, and an Ag/AgCl reference electrode was used. The cyclic voltammograms were obtained by scanning the potential from − 2 to 2 V at a scan rate of 100 mV s^−1^. Transmission electron microscopy (TEM) images obtained with Selmi TEM-125 K microscope at an accelerating voltage of 80.00 kV. The elemental analysis of water-soluble Cd_1-x_Mn_x_Te-alloyed QD samples purified by multiple precipitations was measured by С115М1 atomic emission spectroscopy (AES). EPR spectra were recorded using Х-band EPR spectrometer “Radiopan” at 300 K. One hundred kilohertz modulation of the magnetic field with 0.1 mT amplitude. Visual EPR programs were used for the processing (deconvolution, fitting, and modeling) of the spectra obtained [15]. Powder samples containing QDs were prepared by precipitation of QDs from the aqueous solution by the method described in [16]. The samples were dried at 35 °C for 5 h. Phase composition of the samples was determined by powder XRD measurements using Bruker D8 Advance diffractometer. The identification of the crystalline phase of Cd_1-x_Mn_x_Te was made using the XRD database card: ICSD no. 040413 (Match! software version 3.6.0.111).

## Results and Discussion

### Structural Characterization of the CdTe and Cd_1-x_Mn_x_Te-alloyed QDs

#### TEM Analysis

**Fig. 1:**
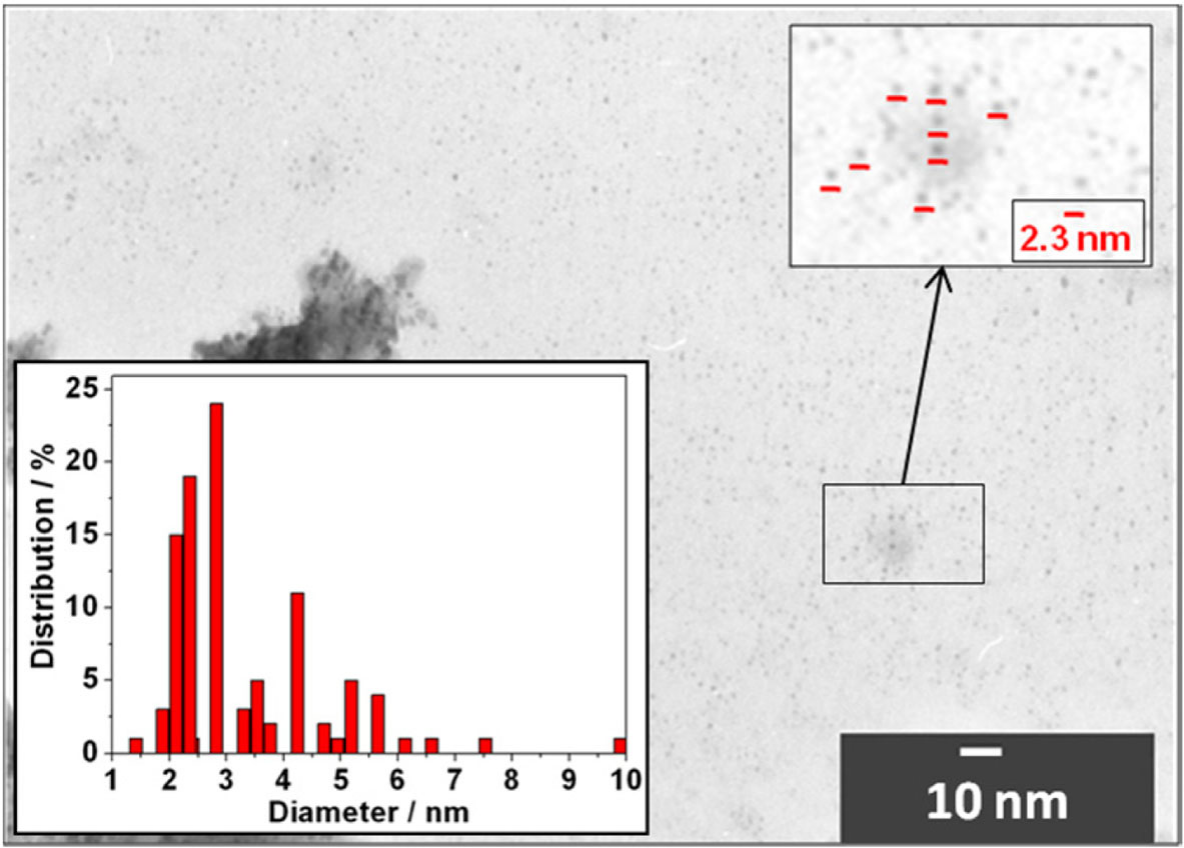
TEM image of Cd_0.91_Mn_0.09_Te-alloyed QDs. Inset: histogram illustrating the Cd_0.91_Mn_0.09_Te-alloyed QD diameter distribution

The TEM images of Cd_0.91_Mn_0.09_Te-alloyed QDs indicated that the average diameter is consistent with the diameter of CdTe QDs calculated from the spectroscopic measurements according to the method described in [14]. Figure 1 shows a TEM image for Cd_0.91_Mn_0.09_Te-alloyed QDs. The vast majority of QDs with an average size of 2.3 ± 0.3 nm were observed. This confirms our assumption that the particles’ size remains unchanged during Mn alloying. Also, larger irregularly shaped objects were observed. It can be seen that those objects are consist of multiple QDs of smaller diameter. Based on these data, we can conclude that bigger objects are aggregates formed during the preparation of the samples for TEM analysis.

### Atomic Emission Spectroscopy Analysis

It was previously shown that only about 20% of the Cd^2+^ ions of the precursor solution participate in the formation of the CdTe QDs by this method of synthesis [13].

The elemental composition of the CdTe and the series of Cd_1-x_Mn_x_Te-alloyed QDs were determined by atomic emission spectroscopy (AES). Cadmium and manganese content was evaluated by the ratio of Cd:Mn (mg/l) for series of Cd_1-x_Mn_x_Te-alloyed QDs with different content of Mn^2+^ ions (Table 1).

**Table 1:** The Cd^2+^/Mn^2+^ ratio in the reaction mixtures (RM) and in the QDs as determined by AES measurements

Sample ID	QDs composition	Ratio Cd^2+^/Mn^2+^ (RM)	Ratio Cd^2+^/Mn^2+^ (QDs/RM)	Ration Cd^2+^/Mn^2+^ (QDs)
1	CdTe	1/0	0.20/0	1/0
2	Cd_0.96_Mn_0.04_Te	1/0.01	0.20/0.01	0.96/0.04
3	Cd_0.97_Mn_0.03_Te	1/0.05	0.20/0.05	0.97/0.03
4	Cd_0.95_Mn_0.05_Te	1/0.10	0.20/0.10	0.95/0.05
5	Cd_0.92_Mn_0.08_Te	1/0.15	0.20/0.15	0.92/0.08
6	Cd_0.91_Mn_0.09_Te	1/0.20	0.20/0.20	0.91/0.09

It is interesting to note that Mn alloying has occurred unevenly with the increasing Mn^2+^ concentration in the reaction mixtures. It is clearly seen that addition at about 1% Mn^2+^ ions (relative to the content of Cd^2+^ ions in the reaction mixture) to the freshly prepared colloidal solution of CdTe nanoclusters leads to the formation of Cd_0.96_Mn_0.04_Te-alloyed QDs. On the other hand when the concentration of added Mn^2+^ was 5%, the formation of Cd_0.97_Mn_0.03_Te-alloyed QDs was observed. This disagreement may suggest that Mn-alloying process is more efficient in the presence of a minor excess of the alloying component. A further addition of 10, 15, and 20% Mn^2+^ ions leads to a consistent Mn alloying with CdTe QDs.

### Spectroscopic Characterization

**Fig. 2:**
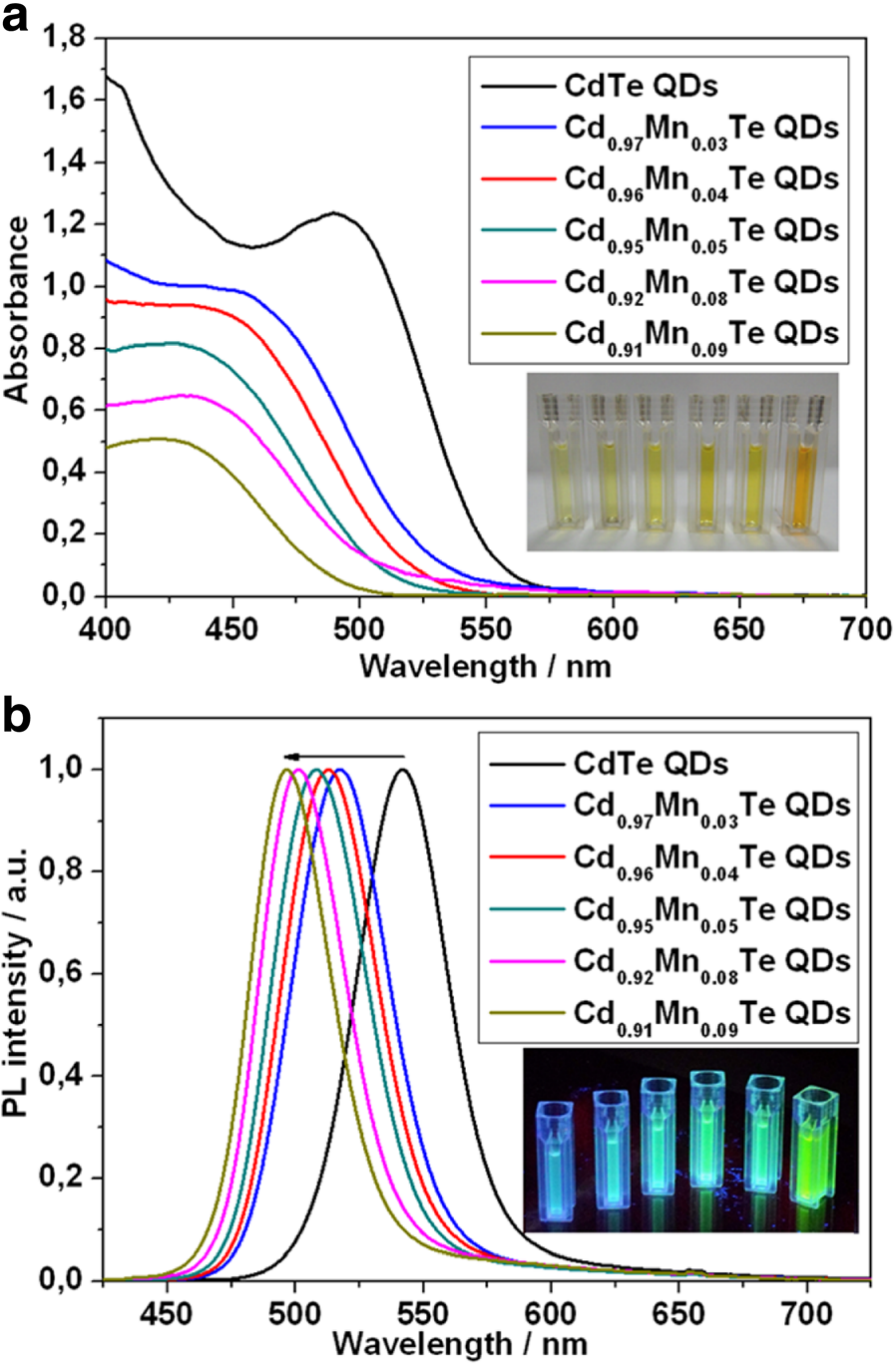
Absorption (**a**) spectra of CdTe and series of Cd_1-x_Mn_x_Te-alloyed QDs with different content of Mn^2+^ ions. Inset: the images of CdTe and series of Cd_1-x_Mn_x_Te-alloyed QDs under daylight-lamp light. Normalized PL (**b**) spectra of CdTe and series of Cd_1-x_Mn_x_Te-alloyed QDs with different content of Mn^2+^ ions. Inset: the images of CdTe and series of Cd_1-x_Mn_x_Te QDs under UV light

The optical properties of the prepared CdTe and Cd_1-x_Mn_x_Te-alloyed QDs were studied by means of Vis region absorption and fluorescence spectra. Figure 2 illustrates a typical absorption (a) and PL spectra (b) of CdTe and series of Cd_1-x_Mn_x_Te-alloyed QDs. Depending on the incorporated Mn^2+^ into CdTe, we observed a hypsochromic shift of the absorption peaks to the shorter wavelength. Also, the blue shift of the PL peaks from 542 to 496 nm was observed. There is a certain mismatch of hypsochromic shift of absorption and PL peaks for the samples 2 (red line) and 3 (blue line) which is probably caused by irregular Mn alloying.

It should be noted that the fluorescence intensity of the Cd_1-x_Mn_x_Te-alloyed QDs decreased with increasing of Mn^2+^ ion content (Additional file 1: Figure S1). It can be explained by the partial conjunction of Mn^2+^ ions that do not participate in alloying process and presence of a stabilizer (thioglycolic acid), which can quench QDs fluorescence [17].

### Cyclic Voltammetric Characterization

A cyclic voltammetry (CV) method was applied to understand band structure changes as the result of the transformation of CdTe QDs into Cd_1-x_Mn_x_Te-alloyed QDs due to the increasing of Mn^2+^ content.

**Fig. 3:**
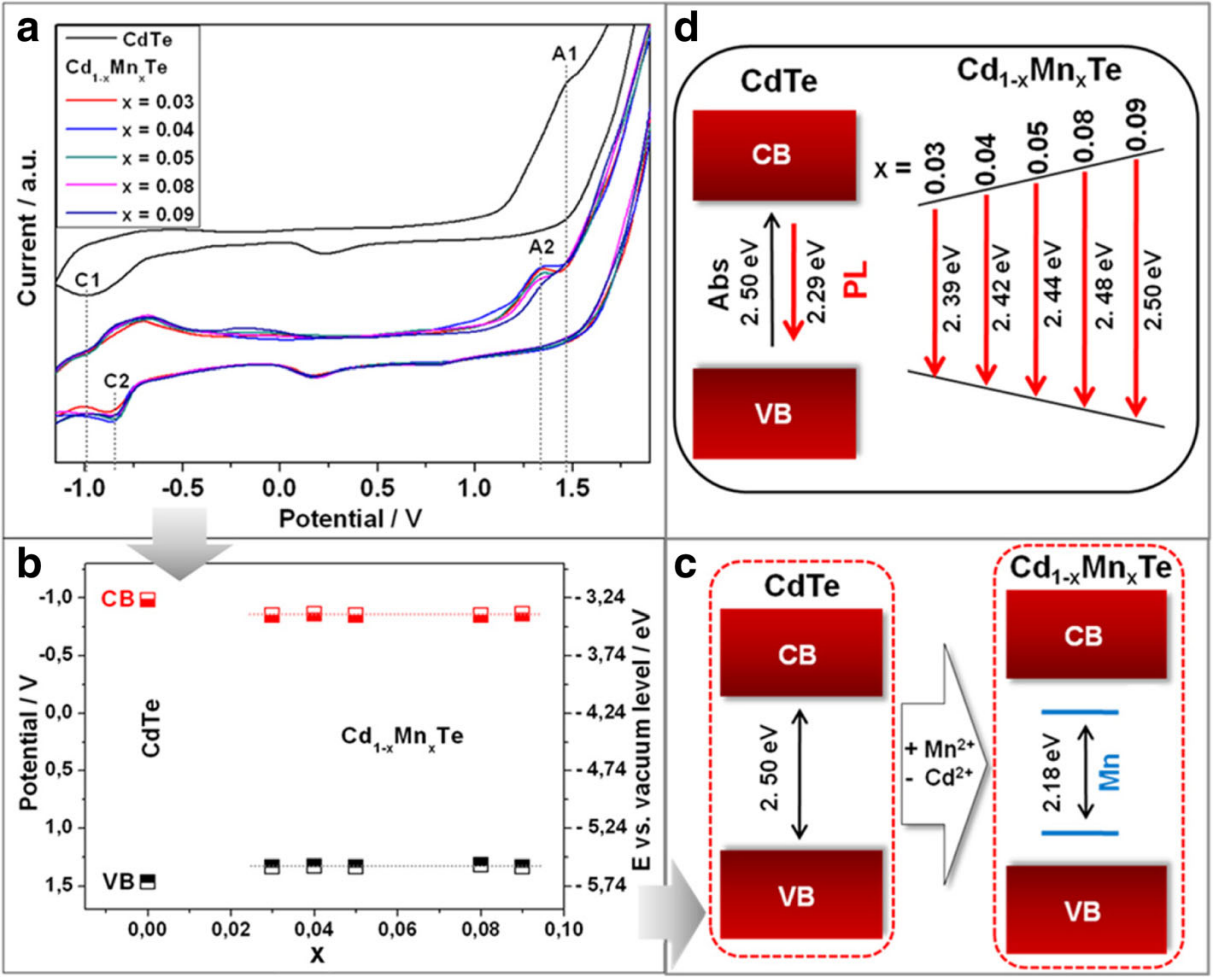
Cyclic voltammograms of colloidal CdTe and series of Cd_1-x_Mn_x_Te-alloyed QDs with different Mn^2+^ ions content (**a**). Plot of valence (VB) and conduction (CB) band edge positions for CdTe and the series of Cd_1-x_Mn_x_Te-alloyed QDs, obtained from the respective anodic (A) and cathodic (C) peak positions (**b**). Scheme of the electrochemical (**c**) and optical (**d**) signal formation

On typical CV of colloidal CdTe QDs, we observed (Fig. 3a) cathodic and anodic peaks at − 1.00 V (marked as C1) and 1.48 V (marked as A1), respectively. The bandgap energy value of 2.48 eV, calculated according to the method described in [18], agrees well with the optical band gap of 2.50 eV obtained from the absorption peak maxima.

Basing on the optical properties of Cd_1-x_Mn_x_Te-alloyed QDs, we expected to observe oxidation at more positive potentials and reduction at more negative potentials for Cd_1-x_Mn_x_Te-alloyed QDs, with increasing of Mn^2+^ content in Cd_1-x_Mn_x_Te-alloyed QDs due to increasing of band gap energy. However, the separation between oxidation peak A2 and reduction peak C2 is too small to correlate with the band gap energy calculated from photoluminescence spectra. Interestingly, the potential difference of 2.18 V between C2 and A2 is absolutely identical for the whole series of Cd_1-x_Mn_x_Te-alloyed QDs samples (Fig. 3a, b).

As shown by Beaulac et al., excitonic PL decay times as long as 5 μs were observed for colloidal Cd_1-x_Mn_x_Se (*x* = 0.004 ± 0.002) QDs (d ≈ 2.2 nm) at 293 K, which arise as a result of thermal equilibrium between the CdSe excitonic states and the very long-lived ligand-field excited state of the Mn^2+^ dopants. Therefore, Mn doping does not quench the excitonic PL of Cd_1-x_Mn_x_Se QDs. Instead, itself-quenching effects of Mn^2+^ PL by thermally assisted back-energy transfer to the Cd_1-x_Mn_x_Se QDs excitonic states take place. [4].

We can assume that in the case of colloidal Cd_1-x_Mn_x_Te-alloyed QDs (d ≈ 2.3 nm) at room temperature, a very similar phenomenon occurs. Using the CV method, the electrochemical activity of “dark” manganese energy levels inside the Cd_1-x_Mn_x_Te-alloyed QDs band gap was detected (Fig. 3b, c). Optical properties of Cd_1-x_Mn_x_Te-alloyed QDs show an increase of the band gap energy with increase of Mn^2+^ content as result of back-energy transfer phenomena (Fig. 3d).

### XRD Analysis

Additional file 1: Figure S2 shows the XRD spectra for CdTe and series of Cd_1-x_Mn_x_Te-alloyed QD dried samples which were precipitated from an aqueous solution using isopropyl alcohol.

The XRD spectrum for CdTe QDs scanning over the two theta range of 20°–60° shows diffraction peak at 25°, which is assigned to the (111) crystal planes of CdTe with cubic crystalline structure [19]. This peak is significantly wider than that of the bulk materials due to the small size of QDs with narrow size distribution. The signal on XRD patterns of all series of Cd_1-x_Mn_x_Te-alloyed QDs is shifted toward higher angles. The peak at 30°–35° can be deconvoluted into two peaks at 30° and 35°, which are assigned to the (200) and (220) planes of Cd_1-x_Mn_x_Te alloy. These results may indicate the formation of Cd_1-x_Mn_x_Te with cubic structure. Such XRD data confirm that the CdTe QDs underwent the Mn-alloying process successfully. Notably that peak centered at 25° decays on XRD pattern of Cd_0.97_Mn_0.03_Te alloyed QDs and vanishes on the XRD patterns of the samples with higher Mn^2+^ content. In the case of Cd_0.97_Mn_0.03_Te QDs sample, we concluded formation of core/shell CdTe/Cd_1-x_Mn_x_Te QDs where the signal of Cd_1-x_Mn_x_Te shell screens the signal of CdTe core. For all subsequent samples, one broad diffraction peak at 30°–35° can testify the further Mn alloying of CdTe QDs and formation of a thicker Cd_1-x_Mn_x_Te shell. Generally, this leads to the formation of QDs with higher manganese contents.

### EPR Measurements

**Fig. 4:**
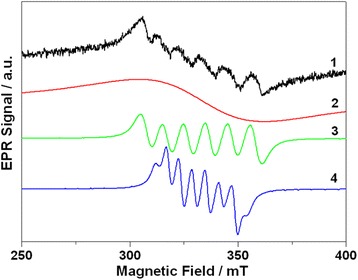
EPR spectrum of Cd_0.97_Mn_0.03_Te-alloyed QDs and its deconvolution: 1—experimental spectrum; 2—Gaussian curve with peak-to-peak linewidth 50 mT. 3—sextet with splitting of ~ 10 mT. 4—sextet with splitting of ~ 6 mT. Details see in the text

The EPR spectra of Cd_0.97_Mn_0.03_Te-alloyed QDs are shown in Fig. 4. The spectrum consists of six asymmetric lines that are superimposed on the broad underlying signal. The presence of six lines in the spectrum is typical for Mn^2+^ ions in the disordered systems [2, 20–22]. However, the shape of the spectrum is more complex than the trivial Mn^2+^-related signal. A detailed analysis has shown that experimental EPR spectrum can be described as a superposition of three signals: broad Gaussian with a linewidth of 50 mT and two sextets with lines spaced by 10 and 6 mT, respectively.

Gaussian-shape EPR signal centered at *g* = 2.0069 (signal **2**) can be attributed to Mn^2+^ ions interconnected by dipole-dipole interaction. These ions are located in regions with a high local concentration of manganese and interact with each other. Due to the lines broadening a strong dipole-dipole interaction between manganese ions masks the specific spectral features being characteristic for isolated manganese ions and leads to a single-line EPR signal. The two other EPR signals (marked as **3** and **4**) should be assigned to isolated Mn^2+^ ions. To determine the parameters of isolated Mn^2+^ ions and its location the spin-Hamiltonian containing electronic, nuclear Zeeman interaction, hyperfine interaction, and the interaction of the electron spin with the crystal field (zero-field splitting term) was used:

$$ \widehat{H}= g\beta \mathbf{BS}-{g}_{\mathrm{N}}{\beta}_{\mathrm{N}}\mathbf{BI}+A\mathbf{SI}+\sum \limits_{n,m}{b}_m^m{O}_m^m $$

where *β* and *β*_N_ denote the Bohr and nuclear magnetons, respectively; **B** is the external magnetic field; *g* and *g*_N_ are the electron and nuclear g-tensors, respectively; **A** is the tensor of the hyperfine interaction; **S** and **I** are the electron and nuclear quantum-mechanical spin operators, respectively; and *b*_*n*_^*m*^ and *O*_*n*_^*m*^ are the crystal field constants and quantum-mechanical operators, respectively. The values of *g*, *g*_N_, *β*, *β*_N_, and *А* are assumed to be isotropic (that being characteristic for Mn^2+^ ions in II-VI compounds). The set of *b*_*n*_^*m*^ parameters is determined by the surroundings of Mn^2+^ and depends on the symmetry of the ions positions.

The signal **2** can be described by the parameters *g* = 2.0069 and *A* = − 94.5×10^−4^ cm^−4^ that can be assigned to the isolated Mn^2+^ ions located in the position near the surface of QDs. At the same time, signal **3** was found to be characterized by parameters *g* = 2.0069, *A* = − 57.5×10^−4^ cm^−4^ and *b*_*4*_^*0*^ = 27.7×10^−4^ cm^−4^. This set of parameters is typical for Mn^2+^ in cation position (Mn_Cd_) of bulk CdTe crystals.

## Conclusions

The synthesis of blue-emitting Cd_1-x_Mn_x_Te-alloyed QDs of small size was developed. The systematic study of their optical and electrochemical properties has been provided. The blue shift of the PL peaks from 542 to 496 nm during the increasing the Mn^2+^ content in Cd_1-x_Mn_x_Te-alloyed QDs was observed. XRD and EPR analysis confirm the successful replacement of cadmium by manganese ions in the process of synthesis. Colloidal Cd_1-x_Mn_x_Te-alloyed QDs show an increase of the band gap energy with increasing Mn^2+^ content at room temperature as a result of thermally assisted back-energy transfer.

## Additional File

Additional file 1: **Figure S1.** The Mn^2+^ content dependence of the photoluminescence integral intensity and the photoluminescence energy maximum of the CdTe QDs and series of Cd_1-x_Mn_x_Te-alloyed QDs. **Figure S2.** XRD patterns for CdTe QDs and series of Cd_1-x_Mn_x_Te-alloyed QDs. (DOCX 153 kb)

## Abbreviations

CV: Cyclic voltammetry
EPR: Electron paramagnetic resonance
PL: Photoluminescence
QDs: Quantum dots
TEM: Transmission electron microscopy
TGA: Thioglycolic acid
XRD: X-ray diffraction
