# Supplementary material for: Synthesis and Properties of Water-Soluble Blue-Emitting Mn-Alloyed CdTe Quantum Dots
Source: Nanoscale Res Lett. 2018 May 2;13:132. doi: 10.1186/s11671-018-2529-y (PMC5931944; doi:10.1186/s11671-018-2529-y)
Supplement: Supplementary file 1 — Figure S1. The Mn2+ content dependence of the photoluminescence integral intensity and the photoluminescence energy maximum of the CdTe QDs and series of Cd1-xMnxTe-alloyed QDs. Figure S2. XRD patterns for CdTe QDs and series of Cd1-xMnxTe-alloyed QDs. (DOCX 153 kb) [file 11671_2018_2529_MOESM1_ESM.docx]

**Additional file 1**

**
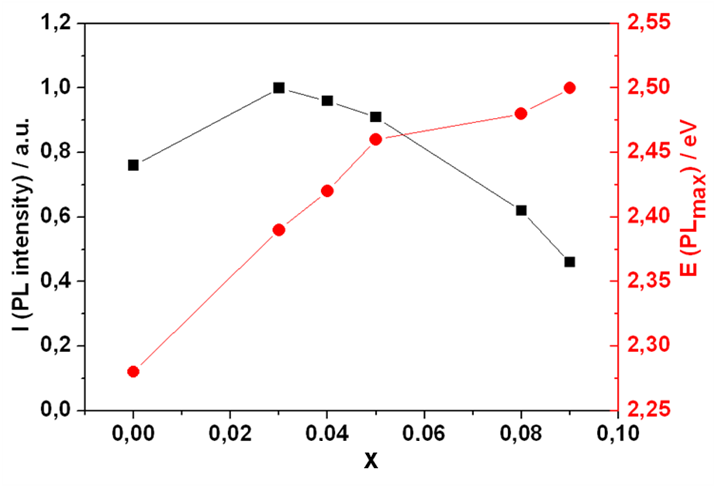
**

**Figure S1** The Mn^2+^ content dependence of the photoluminescence integral intensity and the photoluminescence energy maximum of the CdTe QDs and series of Cd_1-x_Mn_x_Te alloyed QDs.

**
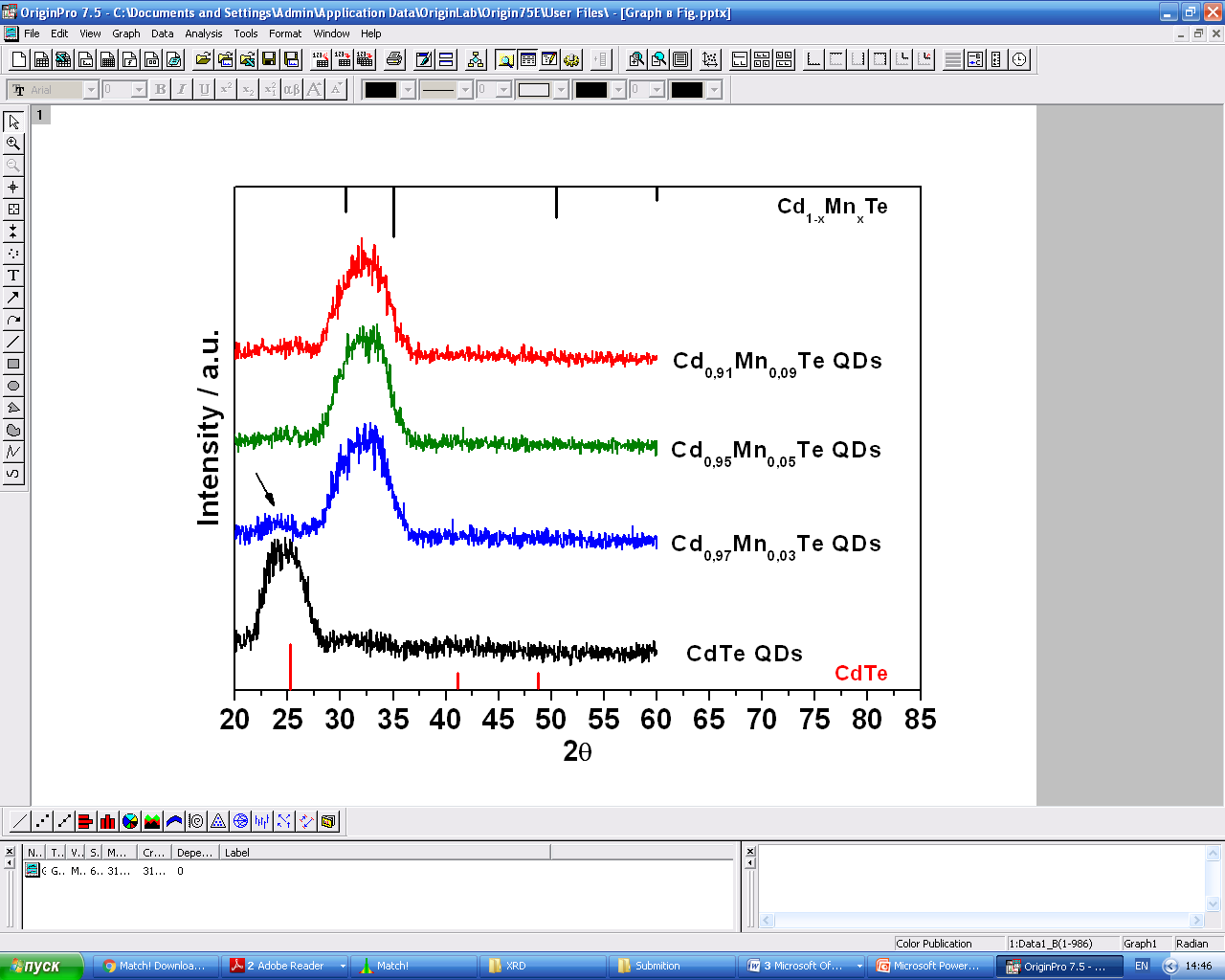
**

**Figure S2** XRD patterns for CdTe QDs and series of Cd_1-x_Mn_x_Te alloyed QDs.
